# The association between opioids, environmental, demographic, and socioeconomic indicators and COVID-19 mortality rates in the United States: an ecological study at the county level

**DOI:** 10.1186/s13690-021-00626-z

**Published:** 2021-06-15

**Authors:** Fares Qeadan, Nana Akofua Mensah, Benjamin Tingey, Rona Bern, Tracy Rees, Erin Fanning Madden, Christina A. Porucznik, Kevin English, Trenton Honda

**Affiliations:** 1 Department of Family and Preventive Medicine, University of Utah, Salt Lake City, UT USA; 2 Department of Family Medicine and Public Health Sciences, Wayne State University, Detroit, MI USA; 3 Albuquerque Area Southwest Tribal Epidemiology Center, Albuquerque, NM USA

**Keywords:** Opioids, COVID-19, Health inequities, Ecological study, Pandemic, Air pollution, Temperature, Mortality rate ratio

## Abstract

**Background:**

The spread of the COVID-19 pandemic throughout the world presents an unprecedented challenge to public health inequities. People who use opioids may be a vulnerable group disproportionately impacted by the current pandemic, however, the limited prior research in this area makes it unclear whether COVID-19 and opioid use outcomes may be related, and whether other environmental and socioeconomic factors might play a role in explaining COVID-19 mortality. The objective of this study is to evaluate the association between opioid-related mortality and COVID-19 mortality across U.S. counties.

**Methods:**

Data from 3142 counties across the U.S. were used to model the cumulative count of deaths due to COVID-19 up to June 2, 2020. A multivariable negative-binomial regression model was employed to evaluate the adjusted COVID-19 mortality rate ratios (aMRR).

**Results:**

After controlling for covariates, counties with higher rates of opioid-related mortality per 100,000 persons were found to be significantly associated with higher rates of COVID-19 mortality (aMRR: 1.0134; 95% CI [1.0054, 1.0214]; *P* = 0.001). Counties with higher average daily Particulate Matter (PM2.5) exposure also saw significantly higher rates of COVID-19 mortality. Analyses revealed rural counties, counties with higher percentages of non-Hispanic whites, and counties with increased average maximum temperatures are significantly associated with lower mortality rates from COVID-19.

**Conclusions:**

This study indicates need for public health efforts in hard hit COVID-19 regions to also focus prevention efforts on overdose risk among people who use opioids. Future studies using individual-level data are needed to allow for detailed inferences.

## Background

The current COVID-19 pandemic has caused significant increases in morbidity and mortality worldwide [1], while also highlighting pre-existing health disparities in many underserved populations [2]. As of July 14, 2020, the World Health Organization (WHO) reported a total of 12,964,809 confirmed cases and 570,288 deaths internationally [1]. Within the same period, the United States recorded 3,407,798 confirmed cases and 136,252 deaths, placing the country at the top for most recorded COVID-19 cases and deaths of any nation [3].

COVID-19 is caused by the severe acute respiratory syndrome coronavirus 2 (SARS-CoV-2) [4]. It is transmitted from person to person through respiratory droplets [5–8] causing respiratory, digestive, and systematic symptoms that may adversely impact health [9]. The risk for serious complications from COVID-19 is magnified with older age, health behaviors like smoking, and underlying health conditions such as diabetes, cancer, cardiac and respiratory conditions [10]. Additionally, researchers have begun to uncover critical insights into how social determinants of health and environmental factors such as temperature and air pollution affect COVID-19 health outcomes. Racial and ethnic inequities in COVID-19 incidence and death in the U.S. are perhaps the most obvious example of the intersection of social marginalization and pandemic outcomes [11]. In cities and states across the U.S., new data show Native American, Hispanic, and Black populations are overrepresented in COVID-19 cases and deaths due to systemic marginalization in housing, income, access to healthcare, education, and professions [12–14]. In terms of environmental factors affecting COVID-19 risk, evidence for the role of temperature in the transmission of coronaviruses is mixed. While some researchers agree that warmer temperatures may result in decreases in coronavirus-related infections [15–17], others have argued that a decline due to increasing temperatures is unlikely [18]. Recent publications using data from the U.S. and China concluded that air pollution, on the other hand, is positively and significantly associated with the number of COVID-19 infections and deaths [19, 20]. There are clear parallels between the underlying health conditions that increase the severity of COVID-19 and the conditions caused by or worsened by long term exposure to air pollutants, as well as connections to environmental racism and residential segregation. These conditions include heart disease, asthma, and compromised lung function, coughing, or breathing difficulties [11, 21–23].

As the pandemic continues to evolve, it is crucial to identify and protect all vulnerable populations whose conditions might be exacerbated by the pandemic. People with opioid use disorders (OUD) are one such group that may require special public health efforts [24]. In a recent publication, the American Medical Association reported that over 35 states had recorded spikes in opioid-related mortality since the beginning of the COVID-19 pandemic [25]. Similarly, researchers assessed the changes to daily numbers of emergency medical services (EMS) encounters for opioid overdose in Kentucky and found that such EMS encounters increased by at least 17% during the 52 days immediately before the COVID-19 state of emergency declaration in early March [26]. Accordingly, health experts have warned that without appropriate measures in place, people with OUD might be disproportionately impacted by the current pandemic [24, 27–30]. Individuals with opioid dependence constitute a vulnerable population in part because the clinical effects of opioid use heighten the risk of COVID-19 infection [24]. Depressed breathing caused by opioid use can result in hypoxemia, which can lead to cardiac, pulmonary, and brain complications [31]. Diminished lung capacity from COVID-19 infection can increase the odds of fatal overdose for persons with OUD [32]. Additionally, many individuals with opioid dependence also have co-morbid conditions that may increase vulnerability to COVID-19. In a study among patients receiving methadone maintenance therapy (MMT) for OUD, researchers found that as many as 83% of participants had more than one comorbid condition, including physical comorbidities such diabetes, cardiac, respiratory disorders and cancers, as well as psychiatric comorbidities [33]. Still, the threat to people with OUD persists well beyond biological susceptibility. Significant risk for those diagnosed with OUD also arises from the social and economic effects of drug criminalization and stigma. Researchers have pointed out that housing instability, interruptions to OUD treatment, increased social isolation due to social distancing measures, and reluctance to seek COVID-19 testing due to stigma associated with drug use have the potential gravely impact people living with OUD [24, 27–30].

This study uses U.S. country-level data to provide a preliminary evaluation of the association between opioid overdose mortality and COVID-19 mortality. Although experts and advocates have highlighted the possibility that COVID-19 has created challenges for people with OUD, the association between risky opioid use and COVID-19 has not been quantified. Given that OUD already causes significant mortality and morbidity each year [34], examining the association between opioid overdose and the COVID-19 pandemic can provide insight into the need for interventions that address opioid use during the COVID-19 pandemic. Additionally, this study examines the impact of environmental, demographic, and socioeconomic variables on COVID-19 mortality.

## Methods

### Setting

All data for this ecological study were collected on the U.S. county-level, as such information is not available on the individual patient-level. Data included all U.S. counties including the District of Columbia; excluded regions included Puerto Rico, the Virgin Islands, Guam, the Northern Mariana Islands, and American Samoa. Data were obtained from several publically available sources for research and reporting. COVID-19 deaths were obtained from the Johns Hopkins University Center for Systems Science and Engineering Coronavirus site [35]. This information was collected as the cumulative count of deaths until June 2, 2020, which was the most up-to-date data at the time of analysis. Opioid-related mortality (2016–2018) and average temperature (2000–2011) data were obtained from the CDC WONDER online multiple cause of death database [36]. Opioid overdose mortality information was identified by ICD-10 codes (underlying cause: X40-X44, X60-X64, X85, Y10-Y14, multiple cause: T40.0–4, T40.6). Opioid-prescription data were obtained from the Opioid & Health Indicators Database (2017) at the American Foundation for AIDS research (amfAR) [37]. Population estimates, age, race, and employment demographics were obtained from the American Community Survey [38]. Particulate Matter (PM_2.5_) exposure data; median household income; health statuses relating to smoking, excessive drinking, access to places of physical activity, and diabetes; unemployment status; and rural status were obtained from the County Health Rankings database (2019) [39]. Median home prices were obtained from the National Association of Realtors (2019) [40]. Hypertension hospitalization rates were obtained from the CDC’s Interactive Atlas of Heart Disease and Stroke database (2015–2017) [41].

### Measurements

The outcome of interest was death due to COVID-19. The raw death count was obtained for each county, and an offset variable of county total population size was used to provide a mortality rate (deaths due to COVID-19 divided by county population size). The primary predictor of interest was the percent of opioid overdose deaths per 100,000 people. Other predictors of interest were the number of opioid prescriptions dispensed per 100 residents, average daily amount of fine particulate matter in micrograms per cubic meter, average maximum temperature (degrees Fahrenheit), population density (total county population size over land area [100 x squared miles]), and percent rural. The air pollution exposure estimate methodology has been described extensively elsewhere [42, 43]. Briefly, daily exposure estimates were derived from the Environmental Protection Agency’s Air Quality System Downscaler model in areas with pollutant monitors, and the Community Multiscale Air Quality model in areas without monitors, at the level of the census tract. County-level exposure estimates were estimated by using the highest censusd tract measured or modeled PM exposure estimate within each county [44]. County demographics controlled for in the analysis included ratio of those 65 years and older over those less than 25 years old, percent of the county population that identifies as Black, percent of the county population that identifies as non-Hispanic white, percent unemployed, log of median household income, log of median home price, and percent of occupations (i.e., health practitioners, sales and office workers, transportation/trucking workers, and education workers) out of total working population 16 and older. Health-related control variables included several known risk factors for COVID-19 morbidity and mortality, inclduing rate of hypertension hospitalizations per 1000 Medicare beneficiaries 65 or older, percent diabetic, percent smokers, percent excessive drinking, and percent with access to places of physical activity. All of the variables were continuous, reflecting rates (or prevalence) at the county-level.

### Statistical analysis

Overall characteristics of counties are presented with means and standard deviations. To provide estimates of variable impact on the rate of COVID-19 mortality (COVID-19 deaths over county population size), an independent multivariable negative-binomial regression model was fit with county-level COVID-19 deaths as the response and all previously mentioned predictors as explanatory variables with percentage of opioid-related mortality as the primary explanatory variable of interest. The total county population size was fit as an offset variable to provide the rate response. Adjusted mortality rate ratios (aMRR) with 95% confidence intervals (CI) and *p*-values are provided. Model fit and diagnostics were assessed. As a sensitivity analysis, the same model was fit except that New York County was removed due to this count having the most extreme number of COVID-19 cases and deaths. Other counties removed one at a time to ensure model fit included Cook County, IL (Chicago), Wayne County, MI (Detroit), and Los Angeles, County, CA (Los Angeles). This prevented skewing associations due to the extremely high death counts in these counties. All hypothesis tests were two-sided with a significance level of 5%. All analyses were conducted in SAS version 9.4 (SAS Institute, Inc., Cary, North Carolina).

## Results

A total of 3142 counties including the District of Columbia were included in the analysis. Table 1 displays the mean and standard deviations of all control variables. Among key risk factors for COVID-19, these data show across all counties, the ratio of those ≥65 years and older to those < 25 years old had a mean of 0.66 (SD = 0.27). The majority of county populations were non-Hispanic white (76.0 [SD = 20.2] white vs. 9.0 [SD = 14.3] Black). Rural residency rates were 59.1 (SD = 32.7). Median household income and median home value were $51,100 (SD = $13,500) and $159,700 (SD = $103,000), respectively. There was an average of 2.7 (SD = 17.9) persons over 100 mile^2^. Average maximum temperature during the study period of March–June 2020 was 65.1 (SD = 9.2) degrees Fahrenheit. Average daily PM_2.5_ exposure (in micrograms per cubic meter) was 9.0 (SD = 2.0). The rate of opioid prescriptions per 100 persons was 61.8 (SD = 32.7) and the opioid mortality rate was 13.7 per 100,000 persons (SD = 9.5).

**Table 1:** Characteristics of counties

Total	3142^a^
Ratio of ≥65 years old to < 25 years old	0.66 (0.27)^b^
% Black	9.0 (14.3)
% Non-Hispanic White	76.0 (20.2)
% Rural	59.1 (32.7)
Median Household Income ($1000)	51.1 (13.5)
Median Home Value ($1000)	159.7 (103.0)
Population Density (persons/100 mile^2^)	2.7 (17.9)
% Unemployed	4.6 (1.7)
% Diabetic	11.6 (2.6)
Hypertension Hospitalizations Rate	6.9 (3.2)
% Smokers	17.9 (3.7)
% Excessive Drinking	17.4 (3.2)
% With access to place of physical activity	62.9 (23.1)
% Health practitioners	5.7 (1.8)
% Sales/office workers	20.5 (2.9)
% Transportation/trucking workers	17.0 (6.0)
% Education workers	9.5 (2.5)
Average summer temperature (°F)	65.1 (9.2)
Average Daily PM_2.5_ (*μ*g/m3)	9.0 (2.0)
Opioid Prescribing Rate per 100 persons	61.8 (32.7)
Opioid Mortality Rate per 100,000 persons	13.7 (9.5)

^a^ all U.S. counties including District of Columbia; ^b^mean (S.D.)

**Fig. 1:**
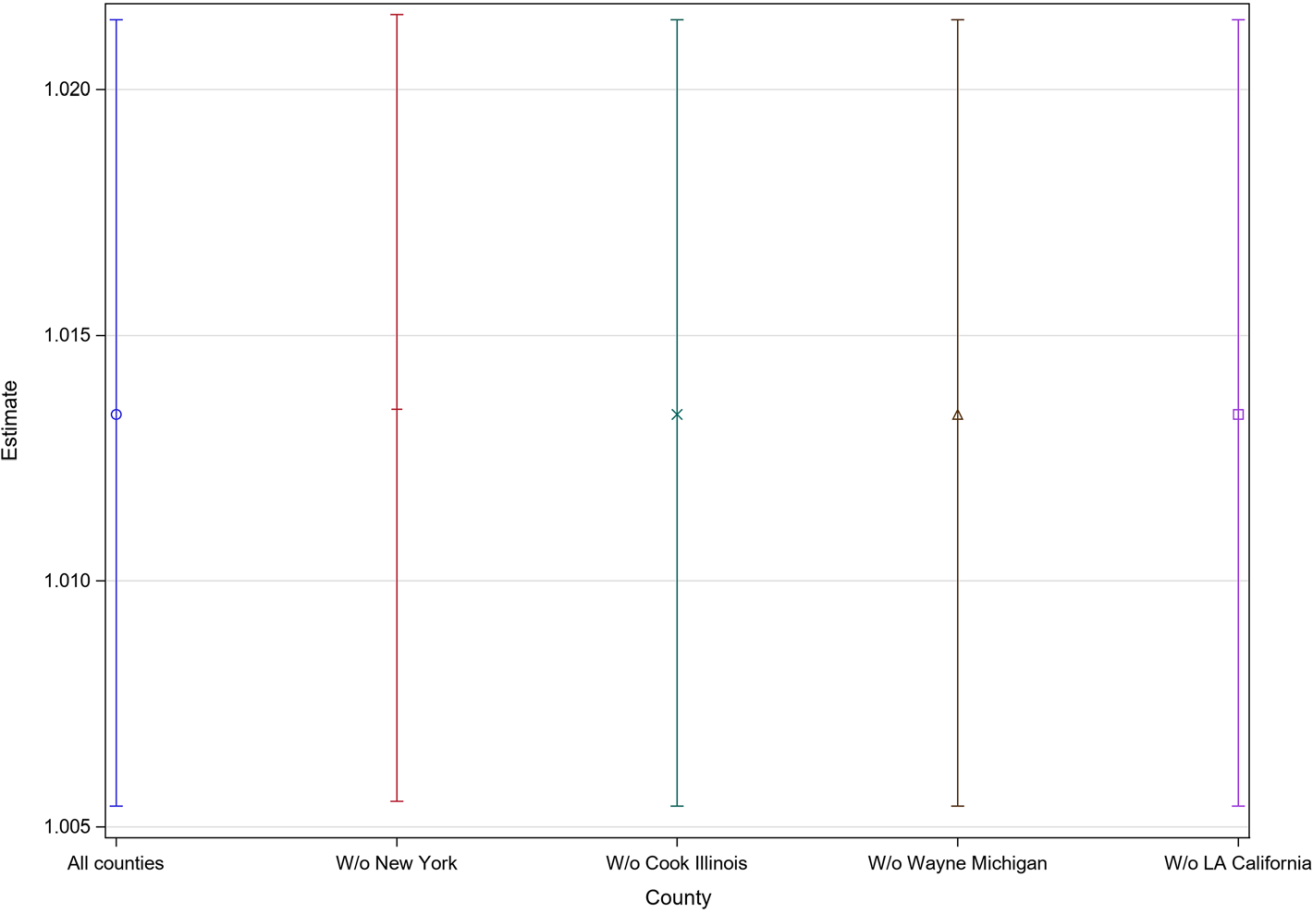
Mortality rate ratios (with 95% CI’s) of “Opioid Mortality Rate per 100,000 persons” on COVID-19 mortality upon removal of outlier counties

Table 2 reports the aMRRs of each variable’s impact on the expected rate of COVID-19 mortality across U.S. counties. Counties with higher rates of opioid overdose mortality per 100,000 persons also saw significantly higher rates of COVID-19 mortality (aMRR: 1.0134; 95% CI [1.0054, 1.0214]). Counties with higher percentages of Black residents exhibited significantly higher rates of COVID-19 mortality (aMRR: 1.0323; 95% CI [1.0255, 1.0390]), whereas counties with higher percentages of non-Hispanic white residents saw significantly lower rates (aMRR: 0.9828; 95% CI [0.9767, 0.9889]). Counties with more rural residents had significantly lower rates of COVID-19 mortality. Counties with higher median household income, population density, and unemployment also displayed significant increases in COVID-19 mortality. Regions with higher percentages of people living with diabetes and grater shares of the workforce in transportation/trucking demonstrated increases in COVID-19 mortality that were on the border of significance. The analysis also showed that as county average maximum temperatures increased, COVID-19 mortality decreased significantly (aMRR: 0.9784; 95% CI [0.9682, 0.9889]). Finally, counties with higher average daily PM_2.5_ exposure exhibited significantly higher rates of COVID-19 mortality (aMRR: 1.0695; 95% CI [1.0194, 1.220]). The results of the sensitivity analyses showed the same associations without New York, Cook, Wayne, and Los Angeles counties (Fig. 1).

**Table 2:** Adjusted estimates of variables impact on COVID-19 mortality^a^

Variables	Adjusted MRR^b^ (95% CI)	*P*-value
Opioid Mortality Rate per 100,000 persons	**1.0134 (1.0054, 1.0214)**	**0.001**
Opioid Prescribing Rate per 100 persons	1.0005 (0.9979, 1.0031)	0.69
Ratio of ≥65 years old to < 25 years old	1.0564 (0.8082, 1.3809)	0.69
% Black	**1.0323 (1.0255, 1.0390)**	**< 0.001**
% Non-Hispanic White	**0.9828 (0.9767, 0.9889)**	**< 0.001**
% Rural	**0.9951 (0.9917, 0.9986)**	**0.01**
log (Median Household Income)	**6.2034 (3.5170, 10.9419)**	**< 0.001**
*log (Median Home Value)*	*0.7907 (0.6062, 1.0332)*	*0.09*
Population Density (persons/100 mile^2^)	**1.0050 (1.0019, 1.0082)**	**0.002**
% Unemployed	**1.0591 (1.0008, 1.1208)**	**0.047**
*% Diabetic*	*1.0414 (0.9948, 1.0904)*	*0.08*
Hypertension Hospitalizations Rate	1.0095 (0.9834, 1.0362)	0.48
% Smokers	1.0030 (0.9708, 1.0363)	0.85
% Excessive Drinking	1.0136 (0.9832, 1.0450)	0.38
% With access to place of physical activity	0.9986 (0.9942, 1.0030)	0.53
% Health practitioners	1.0249 (0.9808, 1.0709)	0.27
% Sales/office workers	1.0083 (0.9797, 1.0379)	0.57
*% Transportation/trucking workers*	*1.0178 (0.9996, 1.0363)*	*0.055*
% Education workers	0.9933 (0.9608, 1.0269)	0.69
Average maximum temperature (°F)	**0.9784 (0.9682, 0.9889)**	**< 0.001**
Average Daily PM_2.5_ (*μ*g/m3)	**1.0695 (1.0194, 1.220)**	**0.01**

^a^ Negative binomial regression model, R^2^: 0.79, Chi-squared Goodness of Fit p-value: 0.70; ^b^ Mortality rate ratio

## Discussion

The present study examined the association between fatal opioid overdose mortality and COVID-19 mortality rates using county-level data. Our results revealed a positive association between these causes of mortality after adjusting for relevant county-level health, demographic, socioeconomic, and environmental predictors, including average daily PM_2.5_ and average maximum temperatures. Specifically, we found that a unit increase in the opioid mortality rate was significantly associated with a 1.3% increase in the COVID-19 mortality rate. Although we observed a similar positive association for county-level opioid prescribing rates, our estimate was not significant. Additionally, we found that an increase of 1 μg/m3 in the average PM_2.5_ resulted in a 7% increase in the COVID-19 mortality rates. In contrast, a rise in 1 °F in the average maximum temperature was associated with a roughly 2% decrease in COVID-19 death rates during this season. Other predictors, such as the percentage of Black residents in a county, median household income, population density, the percentage of unemployed persons in a county, were all positively associated with COVID-19 death rates (aMRR: 1.0323; 95% CI [1.0255, 1.0390], 6.2034; 95% CI [3.5170, 10.9419], 1.0050; 95% CI [1.0019, 1.0082], 1.0591; 95% CI [1.0008, 1.1208], respectively). Counties with greater percentages of rural residents and non-Hispanic white residents, on the other hand, corresponded to significantly lower COVID-19 death rates. Other known individual-level risk factors for COVID-19 deaths and complications, such as the percentage of individuals living with diabetes, the percentage of smokers, and hypertension hospitalization rates, were not statistically significant.

Our finding that increased opioid overdose mortality rates were associated with increased COVID-19 mortality rates, while limited by the ecological nature of the data, is consistent with expert opinions that describe direct and indirect ways individuals with OUDs may be more susceptible to COVID-19 and its devastating impacts [24, 27–29, 45]. This study is one of the first to quantify the association between county-level COVID-19 and opioid-related deaths, and provide preliminary evidence that the warnings of substance use experts appear to be justified. A recent commentary noted that individuals with substance use disorders, including people with OUDs, might be prone to contracting COVID-19 due to the direct effects of substance use on respiratory health [24]. The use of opioids has been associated with breathing disorders that can result in further cardiac, pulmonary or brain-related ailments [31, 46]. Moreover, many individuals with OUD suffer from co-morbid conditions (e.g., heart disease, respiratory disease, cancer, diabetes etc.) [47–49] that might increase the likelihood of COVID-19 infection and death [29, 50].

The majority of the risk to people with OUD during the pandemic may originate from indirect factors such as limited or fragmented treatment options, housing difficulties, and increased social isolation. Because of social distancing measures, many individuals with OUDs may experience limited treatment options or interruptions in care [24, 27]. Emergency departments may prioritize COVID-19 patients leaving patients experiencing drug overdose without adequate emergency medical care [24, 28, 45]. Many individuals with substance use disorders also face unstable housing conditions, further limiting their ability to engage in appropriate physical distancing measures, which may also increase risk of COVID-19 infection. However, those able to undertake physical distancing may experience more social isolation, which might trigger relapse among patients in recovery [24, 45]. Finally, physical distancing may mean more fatal overdoses occur without the presence of observers who could utilize naloxone to reverse the effects of opioids [24].

Although we observed a positive association between opioid prescription rates and COVID-19 mortality rates, the estimate was not statisticaly significant. This may be due in part to the fact that county-level opioid prescription rates largely reflect licit opioid prescriptions, and do not directly capture diverted prescription opioids, or capture the illicitly manufactured opioid supply at all. Moreover, similar high prescribing rates in many counties and little variability in our data might have obscured the actual effects of opioid prescribing practices on COVID-19 mortality. Nevertheless, our results suggest the concern among some physicians that opioid users are at increased risk due to COVID-19 is not without merit [24] and demonstrate a need for measures to ensure that the impact of COVID-19 on individuals who use opioids is minimized.

In our model, we accounted for critical environmental factors that may influence COVID-19 health outcomes. We found significant associations between county-level COVID-19 mortality and both PM_2.5_ and mean maximum temperatures. While higher PM_2.5_ concentration was associated with increased COVID-19 mortality, higher temperatures were associated with lower COVID-19 mortality. In a recent publication, researchers reported an 8% increase in COVID-19 death rate for each 1 μg/m3 rise in PM_2.5_ concentration, markedly similar to our estimate of a 7% increase in COVID-19 death rates. However, this prior study did not find significant estimates for their temperature measures [19]. Similarly, data from several Chinese cities indicated significant positive associations between confirmed COVID-19 cases and five air pollutants including PM_2.5_ [20].

The results of this county-level analysis conform to prior studies that have linked air pollution to poor health outcomes amidst infectious disease outbreaks [51, 52]. Researchers believe that PM_2.5_ exposure, which has been linked to poor cardiovascular and respiratory health outcomes, may increase the likelihood of COVID-19 complications and death [19]. Other experts have pointed to the suppression of the immune system response due to air pollution exposure, which may lead to further complications [53, 54].

Consistent with previous studies, our results also suggest that warmer temperatures may result in the decline of COVID-19 deaths. These prior studies reported higher survival odds of coronaviruses in lower temperatures and diminished viral survival with increasing temperatures [15–17]. However, a recent study by Xie et al., reported findings contradictory to our results. Their research found a positive non-linear association between newly confirmed COVID-19 cases and average temperature. They noted that this association plateaued at 3 °C (i.e. 37.4 F), suggesting that COVID-19 cases are unlikely to decrease with warmer temperatures [18]. Given these mixed results about the association between temperature and COVID-19, one could theorize that there may be a U shaped curve – when it is cold people are indoors more, and when it is very hot people are indoors in air conditioning. So, the association could be understool as less about temperature and the virus than about comfortable ambient temperature for humans. Thus, we do not expect the found temperature association in this study to be consistent throughout the year especially that temperature mortality associations are usually non-linear.

Our findings should be interpreted within certain limitations. First, because we used county-level data, the study may be subject to ecological fallacy. As such, our findings should not be interpreted on the individual patient-level. Additionally, because of the cross-sectional nature of our study, we are unable to establish longitudinal or causal effects. Finally, the dynamic nature of the COVID-19 pandemic implies that our results only reflect the situation up until the time of our analysis. Despite these limitations, our study has some unique strengths. First, we accounted for an extensive list of potential confounders, including the number of opioid prescriptions dispensed per 100 residents, average daily amount of PM_2.5_, average and maximum temperature. We also utilized publicly available data, which allows for straightforward replication and expansion of our work. As more data becomes available, future studies should replicate this study using individual-level data to allow for individual-level inferences.

## Conclusion

Our study highlights the need to protect an already vulnerable population of people with OUD during the COVID-19 pandemic, while emphasizing the potential impact of air pollution and temperature on risk for COVID-19 mortality. Provisions should be made to ensure stable housing and minimal disruptions in care for those individuals with OUD. Further, our research also justifies the need to improve air quality by enforcing current regulations and implementing new rules as needed.

## Data Availability

The datasets during and/or analysed during the current study available from the corresponding author on reasonable request.

## Abbreviations

aMRR: Adjusted mortality rate ratio
PM2.5: Particulate matter (PM) with a diameter of less than 2.5 μm
WHO: World Health Organization
SARS-CoV-2: Severe acute respiratory syndrome coronavirus 2
OUD: Opioid use disorders
EMS: Emergency medical services
MMT: Methadone maintenance therapy
amfAR: American Foundation for AIDS research
CI: Confidence intervals
